# Adipocyte ADAM17 plays a limited role in metabolic inflammation

**DOI:** 10.1080/21623945.2020.1814544

**Published:** 2020-09-06

**Authors:** Joseph C. Lownik, Jared S. Farrar, Janina V. Pearce, Francesco S. Celi, Rebecca K. Martin

**Affiliations:** a Center for Clinical and Translational Research, Virginia Commonwealth University School of Medicine, Richmond, VA, USA; b Department of Internal Medicine, Division of Endocrinology, Diabetes and Metabolism, Virginia Commonwealth University School of Medicine, Richmond, VA, USA; c Department of Microbiology and Immunology, Virginia Commonwealth University School of Medicine, Richmond, VA, USA

**Keywords:** ADAM17, adipocyte, high-fat diet, metabolic inflammation, protease, TNF

## Abstract

The role of ADAM17, its substrates, and its natural inhibitor has been well studied in the context of inflammation, including metabolic inflammation, with mixed results. Previous studies examining global *Adam17* knockdown models and ADAM17 inhibition using overexpression of endogenous ADAM17 inhibitors have shown improved metabolic health and decreased metabolic inflammation. However, there have been no studies examining the role of adipocyte ADAM17 using *in vivo* models. In this study, we developed an adipocyte-specific *Adam17* knockout model using *Adipoq-Cre*-expressing mice crossed with *Adam17*-floxed mice. Using this model, we show that loss of adipocyte ADAM17 plays no evident role in baseline metabolic responses. Surprisingly, in a state of metabolic stress using high-fat diet (HFD), we observed that adipocyte ADAM17 had little effect overall on the metabolic phenotype as well as inflammatory cell populations. Using whole-body metabolic phenotyping, we show that loss of ADAM17 has no effect on energy utilization both at a baseline state as well as following HFD. However, lastly, using high-parameter flow cytometry, we show that loss of adipocyte ADAM17 alters macrophage and eosinophil populations following HFD. Overall, the studies presented here give more insight into the role of ADAM17 in metabolic responses and metabolic inflammation, specifically in adipocytes.

## Introduction

A disintegrin and metalloprotease 17 (ADAM17) is a zinc-dependent metalloprotease, also identified as Tumour necrosis factor alpha (TNF) converting enzyme (TACE) for its role as the primary sheddase of TNF from the surface of cells. ADAM17 is ubiquitously expressed and essential for development, making it challenging to study the function *in vivo* as ADAM17 global knockout (KO) mice die shortly after birth [1,2]. However, mice with ~95% of ADAM17 activity abated (ADAM17^ex/ex^) mice are able to survive postnatally. One of the initial phenotypes of these mice was decreased body weight and potentially increased thermogenic (brown) fat utilization, suggesting a role of ADAM17 in metabolism [3].

In 2014, *Matsui et al.* examined mice overexpressing ADAM17 on high-fat diet (HFD) [4]. In this model, the adipose tissue had increased TNF, macrophage infiltration, fibrosis, and inflammatory cytokines [4]. Inverse to this, ADAM17 inhibition with the matrix metalloprotease inhibitor, Marimistat, resulted in decreased weight gain and increased insulin sensitivity on high-fat diet (HFD) [5]. Both of these studies further emphasized ADAM17 as an important targetable molecule in obesity research. Additionally, haploinsufficiency of ADAM17 led to a protective effect in a HFD model with the observation of smaller adipocytes and decreased metabolic inflammation [6]. However, the specific cell type(s) responsible for this protective phenotype remained unknown.

ADAM17 is thought to have a specific effect on adipose tissue biology, particularly through the shedding of delta-like noncanonical notch ligand 1 (DLK1), also known as preadipocyte factor 1 (Pref-1) [7]. Soluble Pref-1 has been shown to inhibit adipocyte differentiation, while the transmembrane form is unable to do so [7]. Additionally, soluble TNF led to macrophage and inflammatory cell infiltration and activation in perigonadal white adipose tissue (pWAT) in mice both on a chow control diet and HFD. However, the role of adipocyte-specific TNF on this phenomenon is unclear.

Tissue inhibitor of metalloproteinase 3 (TIMP3) is the only known endogenous inhibitor of ADAM17 function that is physiologically produced [8]. Several studies have shown the importance of TIMP3 in altering metabolic inflammation following HFD. Loss of TIMP3 led to increased insulin resistance and metabolic inflammation in a HFD model [8]. On the contrary, overexpression of TIMP3 increased both insulin and glucose sensitivities following HFD. Specifically, macrophage-specific TIMP3 decreased metabolic inflammation in a HFD model. As TIMP3 is released in a soluble form, the protective effect of TIMP3 overexpression in these models could be attributable to several cell types within the microenvironment of cells overexpressing TIMP3 [9].

In addition to having natural endogenous inhibitors, ADAM17 also requires other proteins for proper regulation and substrate specificity. Rhomboid 5 homolog 2 (RHBDF2) also known as inactive rhomboid protein 2 (iRhom2), is a polytopic endoplasmic reticulum protein that has recently been shown to be essential for proper ADAM17 trafficking and function [10]. Loss of iRhom2 has been shown to prevent the release of TNF following lipopolysaccharide stimulation, mediated through the loss of ADAM17 activity [11]. Recent studies have shown contradicting results regarding the effect of global iRhom2 deletion on metabolic inflammation following HFD [12,13]. Badenes *et al*. showed that deletion of iRhom2 protected against HFD-induced obesity through increased thermogenesis [13]. However, another study showed that global loss of iRhom2 resulted in increased fat gain as well as increased metabolic inflammation following HFD [12].

Knowing this, we sought to examine the role of adipocyte ADAM17 in metabolism. No studies thus far have specifically examined the role of adipocyte ADAM17 *in vivo*. To do this, we developed adipocyte-specific *Adam17* knockout mice (ADAM17^adipo^) and examined their baseline function as well as metabolic profiling both in chow control diet mice and HFD fed mice. The studies presented here help to clarify the role of ADAM17 in metabolic inflammation and answer the question as to whether adipocyte ADAM17 is involved in the protective effect of ADAM17 inhibition in metabolic inflammation.

## Results

### Loss of adipocyte ADAM17 has no metabolic effect on chow-fed mice

**Figure 1.**
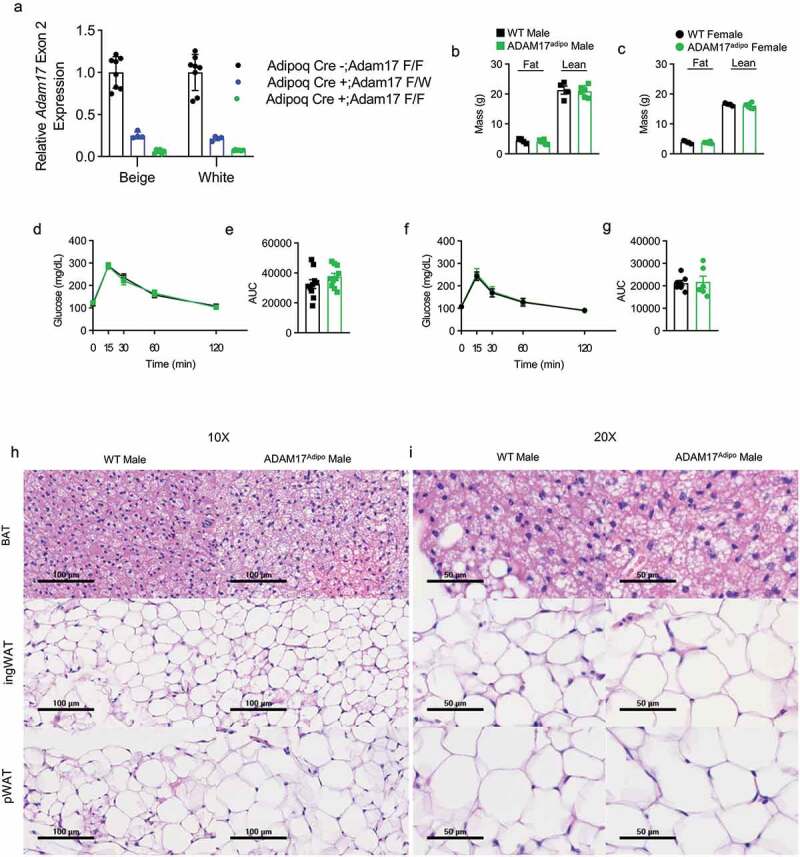
Loss of adipocyte ADAM17 does not have a baseline phenotype. (a) qRT-PCR measurement of relative *Adam17* Exon 2 expression relative *Tbp*. Body composition analysis of 14-week old male (b) and female (c) mice. (d) IPGTT on 14-week old male mice. (e) Statistical analysis of area under the curve (AUC) from (D). (f) IPGTT on 14-week old female mice. (g) Statistical analysis of AUC from (F). 10X (h) and 20X (i) H&E histology of indicated adipose tissue, scale bar indicated on figure. Unpaired student’s t-test (B, C, E, F). Data presented are from one experiment but representative of three independent experiments (B, C, E, F, mean ± SEM). n = 8–18 for each group for IPGTT experiments. n = 4–6 for each group for body composition experiments

To examine the role of adipocyte ADAM17 in metabolic inflammation, we bred *Adam17*flox mice to *Adipoq*-Cre expressing mice to specifically knock ADAM17 out of adipocytes. To check the knockout efficiency of this model, we cultured stromal vascular cells Adam17^fl/fl^ Adipoq-Cre^+^, Adam17^fl/wt^ Adipoq-Cre^+^ and Adam17^fl/fl^ Adipoq-Cre^−^ mice and differentiated them to white adipocytes and beige adipocytes. We then measured the relative expression of Exon 2 of Adam17 which is targeted by the loxP sites in this model. We saw that Exon 2 levels in Adam17^fl/fl^ Adipoq-Cre^+^ adipocytes were >95% reduced relative to Adam17^fl/fl^ Adipoq-Cre^−^ adipocytes while Adam17^fl/wt^ Adipoq-Cre^+^ adipocytes had an intermediate level (Figure 1(a)). As one of the initial findings of ADAM17^ex/ex^ mice was decreased body weight [3], we first examined whether ADAM17^adipo^ mice shared this phenotype. There was no difference in either lean or fat body mass between male (Figure 1(b)) and female (Figure 1(c)) wild-type (WT) and ADAM17^adipo^ mice. Additionally, there was no difference in intraperitoneal glucose tolerance testing (IPGTT) between WT and ADAM17^adipo^ mice for males (Figure 1(d-e)). We saw similar IPGTT blood glucose measurements between WT and ADAM17^adipo^ female mice (Figure 1(f-g)). Also, there were no qualitative differences seen in histology in the brown adipose tissue (BAT), inguinal adipose tissue, or pWAT (Figure 1(h-i)).

**Figure 2.**
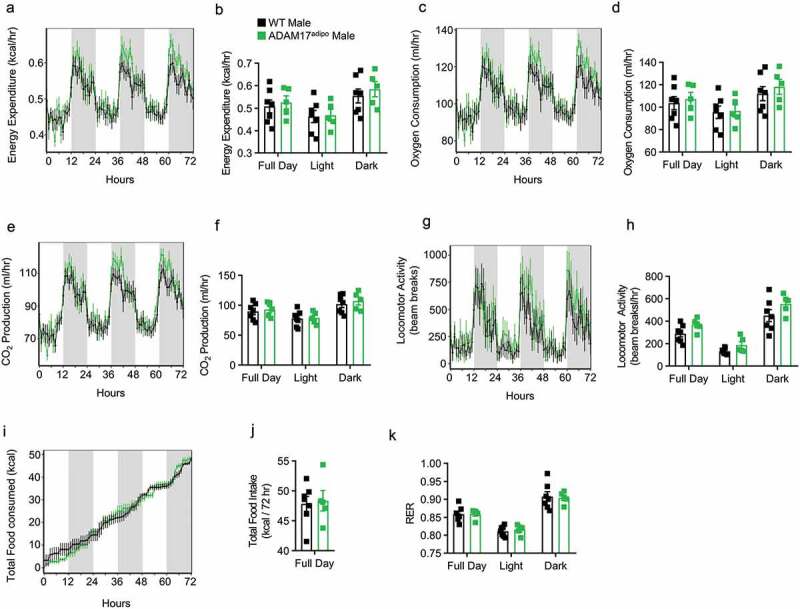
Loss of adipocyte ADAM17 does not affect whole-body metabolic phenotype in male mice. 14-week old male mice were subjected to whole-body metabolic phenotyping using the TSE PhenoMaster system. Total energy expenditure (a-b), oxygen consumption (c-d), CO2 production (e-f), locomotor activity (g-h), caloric intake (i-j) and respiratory exchange ratio (k) were all measured for 72 h Generalized linear regression modelling with regression analysis for body weight (B, D, F, H, J, K). Data presented are from one independent experiment (A – K, mean ± SEM). n = 5–7 mice per genotype

To further characterize the metabolic characteristics of ADAM17^adipo^ mice, we conducted thorough metabolic phenotyping of both male and female ADAM17^adipo^ mice using the TSE PhenoMaster system. We examined 14-week-old chow-fed mice and found no difference between WT and ADAM17^adipo^ male mice for total energy expenditure (Figure 2(a-b)), oxygen consumption (Figure 2(c-d)), CO_2_ production (Figure 2(e-f)), locomotor activity (Figure 2(g-h)), caloric intake (Figure 2(i-j)), and respiratory exchange ratio (RER) (Figure 2(k)). There was also no difference between 14-week-old chow-fed WT and ADAM17^adipo^ female mice for any of the above-mentioned parameters (Supplemental Figs. 1a-k). While there initially appeared to be a difference between WT and ADAM17^adipo^ female mice for caloric intake, regression analysis indicated that this was due to weight differences in the cohort used.

### Loss of adipocyte ADAM17 has minimal metabolic effect following HFD

**Figure 3.**
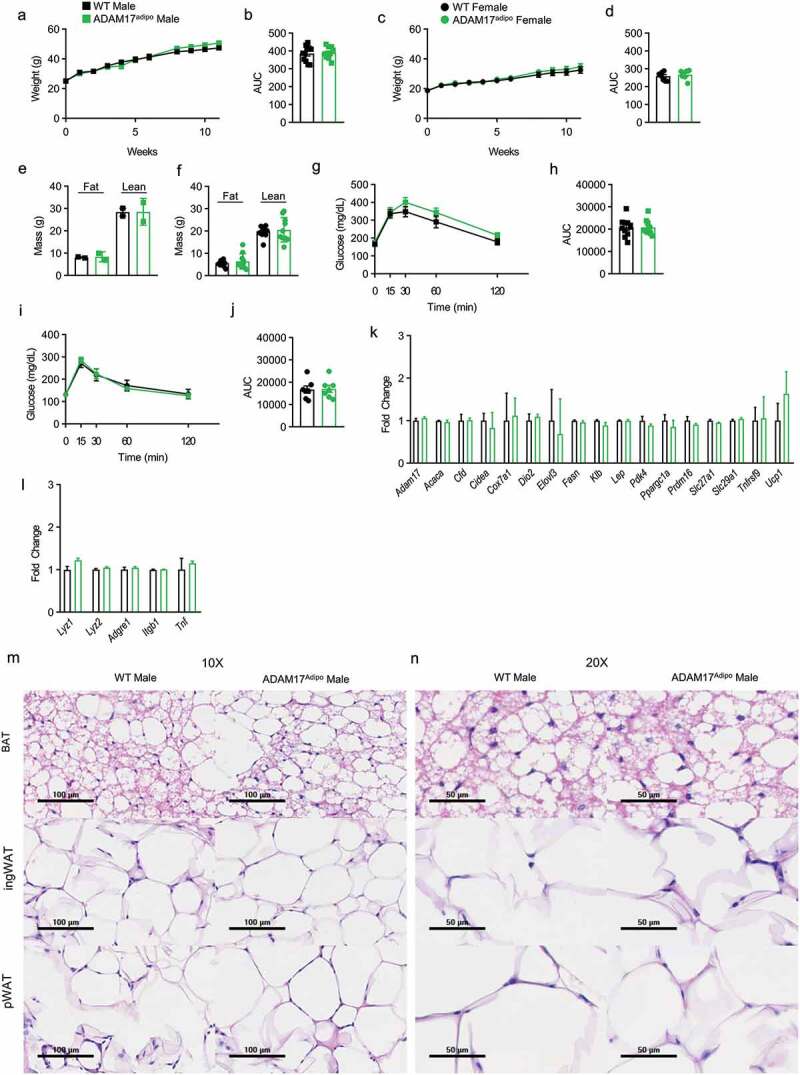
Loss of adipocyte ADAM17 does not affect responsiveness to HFD. (a) Male mice were placed on HFD for 11 weeks and weights were measured weekly. (b) Statistical analysis of AUC from A. (c) Female mice were placed on HFD for 11 weeks and weights were measured weekly. (d) Statistical analysis of AUC from C. Body composition analysis of male (e) and female (f) mice after 14 weeks of HFD diet. (g) IPGTT on male mice following 12 weeks of HFD. (h) Statistical analysis of AUC from G. (i) IPGTT on female mice following 12 weeks of HFD. (j) Statistical analysis of AUC from I. (k) Adipose tissue gene panel from RNA-seq data showing fold change relative to WT for male mice. (l) Inflammatory gene panel from RNA-seq data showing fold change relative to WT for male mice. 10X (m) and 20X (n) H&E histology of indicated adipose tissue, scale bar is indicated on figure. Unpaired Student’s t-test (B, D, E, F, H, J). Data presented are from one experiment but representative of three (a – j) and one (k – l) independent experiments (A-L, mean ± SEM). n = 9–15 for each group for IPGTT experiments. n = 2–8 for each group for body composition experiments. n = 3 for each group for RNA-seq experiments

To examine the role of adipocyte ADAM17 under metabolic stress, we placed WT and ADAM17^adipo^ male and female mice on HFD for 10–14 weeks starting at 8–10 weeks of age. Weight gain was monitored and there was no observable difference between WT and ADAM17^adipo^ mice for both males (Figure 3(a-b)) and females (Figure 3(c-d)). No difference was seen in lean and fat body mass composition between WT and ADAM17^adipo^ mice for both males (Figure 3(e)) and females (Figure 3(f)) after HFD. Following HFD, we conducted an IPGTT and observed no difference between WT and ADAM17^adipo^ mice glucose measurements for both males (Figure 3(g-h)) and females (Figure 3(i-j)).

Following HFD, we conducted RNA-sequencing (RNA-seq) analysis on pWAT tissue samples from WT and ADAM17^adipo^ male mice. No differences in adipocyte genes between WT and ADAM17^adipo^ mice were observed (Figure 3(k)). Correspondingly, we saw no difference in macrophage inflammatory markers between these mice (Figure 3(l)). Similar to what was seen in baseline chow-fed mice, we saw no qualitative differences in BAT, inguinal adipose tissue, or pWAT as measured by H&E histology (Figure 3(m-n)).

**Figure 4.**
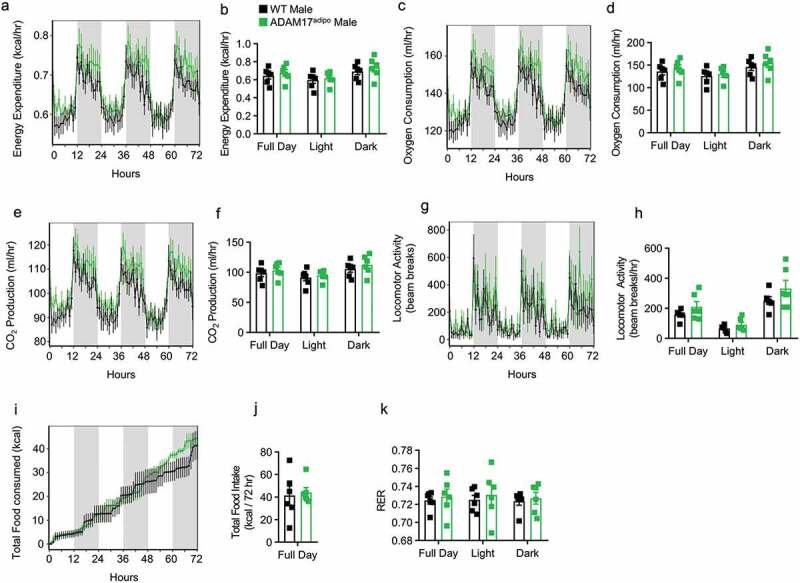
Loss of adipocyte ADAM17 does not affect whole-body metabolic phenotype in male mice following HFD. Following 12 weeks of HFD, male mice were subjected to whole-body metabolic phenotyping using the TSE PhenoMaster system. Total energy expenditure (a-b), oxygen consumptions (c-d), CO2 production (e-f), locomotor activity (g-h), caloric intake (i-j) and respiratory exchange ratio (k) were all measured for 72 h. Generalized linear regression modelling with regression analysis for body weight (B, D, F, H, J, K). Data presented are from one independent experiment (A – K, mean ± SEM). n = 5–7 mice per genotype

Next, we repeated thorough metabolic phenotyping of WT and ADAM17^adipo^ mice following HFD. As was seen in chow-fed mice, no difference was seen between WT and ADAM17^adipo^ mice for total energy expenditure (Figure 4(a-b)), oxygen consumption (Figure 4(c-d)), CO_2_ production (Figure 4(e-f)), locomotor activity (Figure 4(g-h)), caloric intake (Figure 4(i-j)), or RER (Figure 4(k)). Additionally, there was no difference between WT and ADAM17^adipo^ mice for any of the above-mentioned parameters for 14 weeks old chow-fed female mice (Supplemental Figs. 2a-k).

### Immunophenotyping of ADAM17^adipo^ mice following HFD

**Figure 5.**
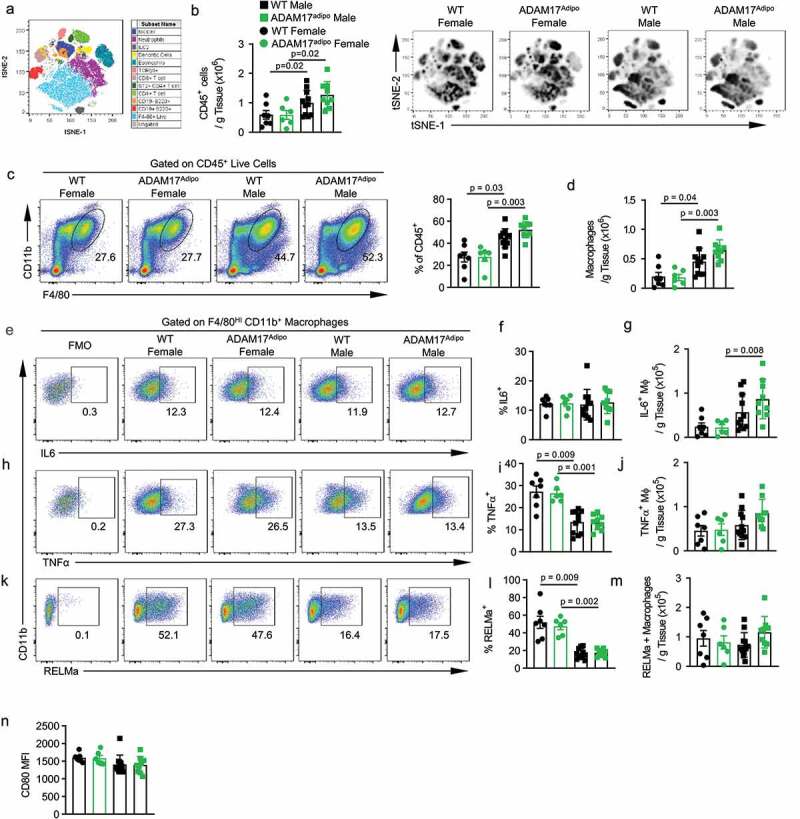
Loss of adipocyte ADAM17 has a small effect on macrophage inflammation following HFD. (a) tSNE analysis pre-gated on CD45^+^ leukocytes in the pWAT following 12 weeks of HFD. (b) Total number of CD45^+^ leukocytes per gram of pWAT tissue following 12 weeks of HFD. (c) Representative pseudocolor flow cytometry plots pre-gated on CD45^+^ live cells gated for macrophages. (d) Statistical analysis of the total number of macrophages per gram of pWAT tissue following 12 weeks of HFD gated from C. (e) Representative pseudocolor flow cytometry plots for intracellular cytokine staining for IL-6 pregated on macrophages following 12 weeks of HFD. Statistical analysis of the relative (f) and absolute (g) number of IL-6^+^ macrophages as gated in E. (h) Representative pseudocolor flow cytometry plots for intracellular cytokine staining for TNF pregated on macrophages following 12 weeks of HFD. Statistical analysis of the relative (i) and absolute (j) number of TNF^+^ macrophages as gated in H. (k) Representative pseudocolor flow cytometry plots for intracellular cytokine staining for RELMa pregated on macrophages following 12 weeks of HFD. Statistical analysis of the relative (l) and absolute (m) number of RELMa^+^ macrophages as gated in K. Statistical analysis of surface CD80 MFI on macrophages following 12 weeks of HFD. – one-way ANOVA with Tukey’s post-test (A, D, F, G, I, J, L, L, N). Data presented are from one experiment but representative of three independent experiments (A, D, F, G, I, J, L, M, N, mean ± SEM). n = 9–15 for each group

To further examine the role of adipocyte ADAM17 in metabolic inflammation, we conducted a thorough immunophenotyping of inflammatory infiltrate in the pWAT using high dimensional, multiparameter flow cytometry (Figure 5(a)). Total leukocytic (CD45^+^) infiltrate into pWAT in HFD fed mice between WT and ADAM17^adipo^ mice was similar for both males and females (Figure 5(b)). Additionally, there were significantly more CD45+ leukocytes in male mice for both genotypes compared to female mice (Figure 5(b)). Macrophage number was similar in ADAM17^adipo^ female mice compared to WT mice (Figure 5(c-d)). There was no difference in macrophages in pWAT of ADAM17^adipo^ male mice compared to WT mice (Figure 5(c-d)). Interestingly, male mice had significantly higher numbers of macrophages compared to female mice for both WT and ADAM17^adipo^ mice (Figure 5(c-d)).

We next examined pro-inflammatory cytokine production by macrophages in these mice. We saw no difference in the relative number of macrophages producing TNF (Figure 5(e-f)) or IL-6 (Figure 5(h-i)) between WT and ADAM17^adipo^ mice for either sex. However, there was a significantly higher relative number of macrophages producing TNF in female mice compared to male mice for both WT and ADAM17^adipo^ strains (Figure 5(e-f)). There was a trend towards an increase in the absolute number of TNF^+^ (Figure 5(g)) as well as IL-6^+^ (Figure 5(j)) macrophages in male ADAM17^adipo^ mice compared to WT mice, but this was not significant. While there was a significant increase in the relative number of TNF producing macrophages in female mice for both genotypes (Figure 5(e-f)), the absolute number of TNF^+^ macrophages in female mice was significantly lower for both genotypes compared to male mice (Figure 5(g)). Additionally, there were significantly fewer absolute IL-6^+^ macrophages in female mice for both genotypes compared to male mice (Figure 5(j)).

We next examined the M2 macrophage marker resistin-like molecule alpha (RELMa). We observed no difference in the relative number of macrophages producing RELMa between WT and ADAM17^adipo^ mice for both males and females (Figure 5(k-l)). However, similar to TNF, the relative number of macrophages producing RELMa was significantly higher in female mice compared to male mice for both genotypes (Figure 5(k-l)). We did not observe a difference in CD80 levels on macrophages between WT and ADAM17^adipo^ mice for both males and females (Figure 5(n)) nor was there a difference in CD80 levels between male and female mice for both genotypes (Figure 5(n)).

**Figure 6.**
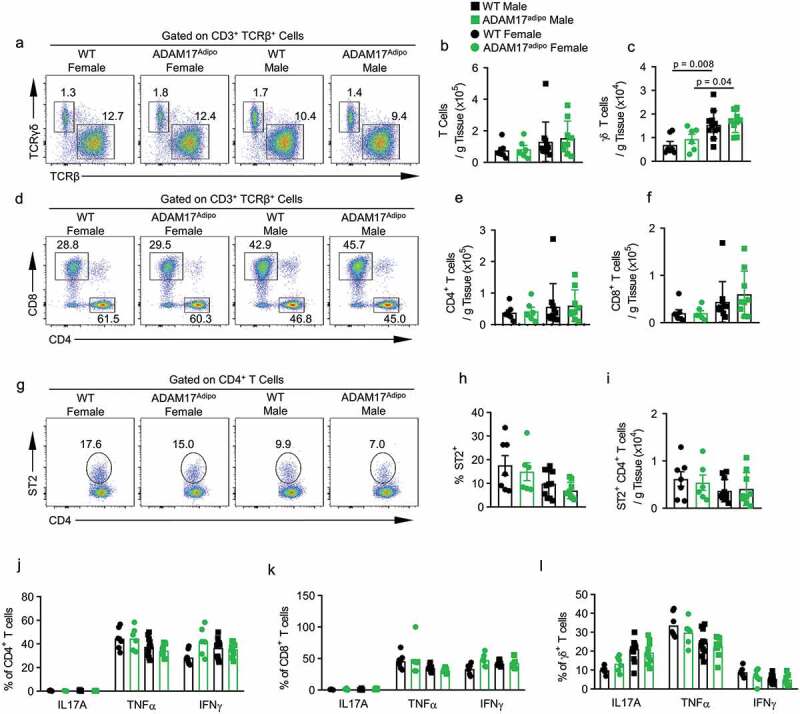
Loss of adipocyte ADAM17 does not affect T cell responses following HFD. (a) Representative pseudocolor flow cytometry plots pre-gated on CD3^+^ T cells. Statistical analysis of the absolute number of ɑβ (b) and ɣδ (c) T cells per gram of pWAT tissue following 12 weeks of HFD gated from A. (d) Representative pseudocolor flow cytometry plots pre-gated on TCRβ^+^ T cells. (e) Statistical analysis of the absolute number of CD4^+^ (E) and CD8^+^ (f) T cells per gram of pWAT tissue following 12 weeks of HFD gated from D. (g) Representative pseudocolor flow cytometry plots pre-gated on CD4^+^ T cells showing ST2 expression. Statistical analysis of the relative (h) and absolute (i) number of ST2^+^ CD4^+^ T cells following 12 weeks of HFD. Percentage of CD4^+^ (j), CD8^+^ (k) and γδ (l) T cells producing indicated cytokines. one-way ANOVA with Tukey’s post-test (B, C, E, F, H – L). Data presented are from one experiment but representative of three independent experiments (B, C, E, F, H – L, mean ± SEM). n = 9–15 for each group

While there was no significant difference in the relative and absolute numbers of αβ T cells between WT and ADAM17^adipo^ mice for both males and females, there were significantly more γδ T cells in the pWAT of male mice for both genotypes compared to female mice (Figure 6(a-c)). There was a trend towards increased numbers of αβ and γδ T cells in male ADAM17^adipo^ mice compared to male WT mice but this was not significant (Figure 6(b-c)). Interestingly, there were significantly increased numbers of both αβ and γδ T cells in male compared to female mice for both genotypes (Figure 6(b-c)). Additionally, we saw no difference in absolute numbers of CD4^+^ and CD8^+^ T cells between WT and ADAM17^adipo^ mice for either sex (Figure 6(d-f)). However, there were significantly more CD4^+^ (Figure 6(e)) and CD8^+^ (Figure 6(f)) T cells in male compared to female mice for both genotypes. As ST2 (ILRL1) expression on CD4^+^ T cells in pWAT has been shown to have a beneficial role in immunometabolic regulation, we examined this cell population and saw no difference between WT and ADAM17^adipo^ mice for both male and females (Figure 6(h-i)). Interestingly, the relative and absolute numbers of ST2^+^ CD4^+^ T cells in females trended higher for both genotypes compared to males, although this was not significant (Figure 6(h-i)). We saw no difference in the relative number of interleukin 17 alpha (IL17A), TNFα, or interferon gamma (IFNγ) producing CD4^+^ T cells (Figure 6(j), Supplemental Figs. 3a-d), CD8^+^ T cells (Figure 6(k), Supplemental Figs. 3e-h), or γδ T cells (Figure 6(l), Supplemental Figs. 4a-d) between WT and ADAM17^adipo^ mice for both male and females.

**Figure 7.**
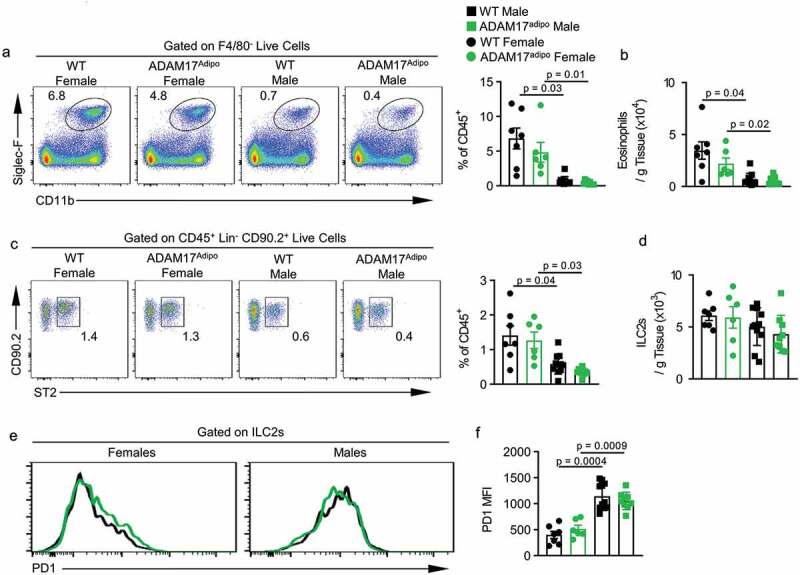
Th2 environment is altered in ADAM17^adipo^ mice following HFD. (a) Representative pseudocolor flow cytometry plots pre-gated on F4/80^−^ live cells. (b) Statistical analysis of the absolute number of eosinophils per gram of pWAT tissue following 12 weeks of HFD gated from A. (c) Representative pseudocolor flow cytometry plots pre-gated on Lin^−^ CD90.2^+^ live cells. (d) Statistical analysis of absolute numbers of ILC2s per gram of pWAT tissue following 12 weeks of HFD gated from C. (e) Representative histograms of PD1 expression on ILC2s from male and female mice following HFD. (f) Statistical analysis of PD1 MFI on ILC2s., one-way ANOVA with Tukey’s post-test (B, D, F). Data presented are from one experiment but representative of three independent experiments (B, D, F, mean ± SEM). n = 9–15 for each group

We saw a trend towards decreased levels of eosinophils in female ADAM17^adipo^ mice compared to female WT mice, but this was not significant (Figure 7(a-b)). However, there were significantly more relative and absolute numbers of eosinophils in female mice compared to male mice for both genotypes (Figure 7(a-b)). As group 2 innate lymphoid cells (ILC2s) have been shown to be important in metabolic homoeostasis, we next examined their levels in the pWAT of these mice [14]. There was no difference in the ILC2 levels between female WT and ADAM17^adipo^ mice (Figure 7(c-d)). However, there was a trend towards fewer ILC2s in the pWAT of male ADAM17^adipo^ mice compared to WT mice, although this was not significant (Figure 7(c-d)). Interestingly, there were significantly more relative and absolute numbers of ILC2s in female mice compared to male mice for both genotypes (Figure 7(c-d)). We then examined the exhaustion marker, programmed cell death protein 1 (PD1), which has been shown to be upregulated in response to TNF in HFD models [15]. We saw no difference in PD1 expression on ILC2s between WT and ADAM17^adipo^ mice for either sex (Figure 7(e-f)). Intriguingly, there was a significant difference between male and female ILC2 PD1 expression for both WT and ADAM17^adipo^ strains with male mice having higher levels of PD1 than females (Figure 7(e-f)).

## Discussion

Several studies have examined the role of ADAM17 and TNF in adipocyte biology as well as metabolic inflammation [8,16,17]. ADAM17 has been studied from near-total knockout to cell-specific knockout levels with intriguing results suggesting diverse roles for ADAM17 on specific cell types in metabolic inflammation [16]. However, to our knowledge, this is the first study examining the role of ADAM17 specifically in adipocytes both at a baseline level and in a stressed metabolic environment during a HFD challenge.

Initial studies examining ADAM17^ex/ex^ mice showed decreased body weight at 8 weeks as well as a potential for improved metabolic health. However, we did not see evidence of decreased body weight in ADAM17^adipo^ mice suggesting that the effect in ADAM17^ex/ex^ mice may not have been adipocyte specific. Intriguingly, ADAM17^±^ mice have decreased fat pad weight as well as an increased number of small adipocytes following HFD compared to WT mice [6]. This protective role was thought to be due to decreased ADAM17 substrate release from adipocytes, including TNF and PREF-1 [7]. With our model examining adipocyte-specific ADAM17 deletion, we were able to conclusively answer the question as to whether ADAM17, specifically on adipocytes, has a role in metabolic inflammation. To our surprise, we saw no difference in lean and fat body mass between WT and ADAM17^adipo^ mice for either sex following HFD. These findings suggest that loss of adipocyte ADAM17 does not have a protective effect following HFD-induced metabolic inflammation that was observed in ADAM17^±^ and TIMP3-overexpression models. Taken together with these previous studies, we hypothesize that the protective effect of the loss of ADAM17 either by haploinsufficiency at a global level or TIMP3 overexpression is not mediated through adipocytes but rather other cell type(s).

Aligning with the lack of overt phenotypic differences between WT and ADAM17^adipo^ mice, we saw only trends in differences in inflammation between these strains following HFD. While it has been shown that male mice have increased weight gain following HFD and decreased glucose tolerance to IPGTT challenge, the inflammatory infiltrate between male and female WT mice was strikingly, and statistically, different. Most notably, we saw significantly higher relative and absolute numbers of eosinophils and ILC2s in female mice following HFD compared to male mice following HFD. Additionally, we saw significant increases in the relative, but not absolute, number of M2 macrophages, as defined by RELMa expression, in female mice compared to male mice. Together, these findings suggest that either a) females have an enhanced Th2 skewed environment in the pWAT following HFD or b) secondary to enhanced weight gain in male mice, male mice eventually lose the protective type 2 (Th2) immune environment that is still remnant in female mice. However, it was intriguing that female mice had increased relative numbers of TNF^+^ macrophages compared to male mice for both genotypes, as TNF is primarily thought to be proinflammatory, yet female mice seem to have decreased overall inflammation. Additionally, we saw significantly lower levels of the ILC2 exhaustion marker PD1 in female mice compared to male mice, despite the elevated relative numbers of TNF^+^ macrophages, suggesting a more complex role for TNF in regulating ILC2 responses and exhaustion.

Overall, these studies shed light on adipocyte ADAM17 function at a baseline level as well as following HFD using a cell-specific knockout model. The findings presented here help to clarify the role of ADAM17 in metabolic inflammation, as most of the models examining this sheddase have not used cell-specific knockouts; those that have used cell-specific knockouts have not examined adipocyte ADAM17. Our findings demonstrate that the previously reported protective effects of ADAM17 inhibition in metabolic inflammation are not a direct result of ADAM17 inhibition on adipocytes but rather other cell types either directly in the microenvironment of adipocytes, or in other organs such as the liver or CNS tissue. Future studies will be needed to further delineate the importance of ADAM17 in metabolic inflammation.

## Methods

### Mice

All animal experiments were performed under the guidelines established by the Institutional Animal Care and Use Committee (IACUC) of Virginia Commonwealth University. Mice were housed in environmentally controlled conditions with a 12-h light/dark cycle (0600 lights on; 1800 lights off) and *ad libitum* food and water. Mice were maintained on Teklad non-irradiated diet 7019. Mice expressing Cre recombinase under the *Adipoq* promoter were purchased from Jackson Laboratory (028020) and crossed to *Adam17* floxed mice also purchased from Jackson Laboratory (009597). *Adam17* floxed mice had previously been backcrossed to a C57Bl6/J background and *Adipoq*-Cre mice were already backcrossed to the same C57Bl/6/J background. Littermate controls were used for all experiments. At experimental endpoint, mice were euthanized by isoflurane inhalation followed by cervical dislocation.

### Stromal vascular isolation, cell culture, and Adam17 measurements

Stromal vascular fraction (SVF) cells were isolated from the inguinal fat pad of a 1 month old male C57BL/6 J mouse as described previously [18]. For white adipocyte differentiation, an induction cocktail comprising basal medium plus 5 μg/mL insulin, 1 μM dexamethasone, 0.5 mM 3-isobutyl-1-methylxanthine (IBMX), and 125 μM indomethacin was used for 2 days. After induction, differentiating white adipocytes were maintained in basal medium and 5 μg/mL insulin for 9–12 days, changing medium every 2 days. For beige adipocyte differentiation, the white adipocyte induction cocktail was supplemented with 0.5 μM rosiglitazone, and 1 nM triiodothyronine (T3). After induction, beige differentiating adipocytes were maintained in basal medium and 5 μg/mL insulin, 1 μM rosiglitazone and 1 nM T3 for 9–12 days, changing medium every 2 days. All chemicals were purchased from Sigma-Aldrich. *Adam17* Exon 2 and *Tbp* qRT-PCR was conducted as previously described [19]. Primers are as follows: *Adam17* Exon 2 Forward 5ʹ-GCTCTCAGACTACGACATCCT-3ʹ, *Adam17* Exon 2 Reverse 5ʹ- GTCGCAGACTGTAGATCCCTT-3ʹ, *Tbp* Forward 5ʹ- TTCCAAAACTCCGGGTAGGC-3ʹ, *Tbp* Reverse 5ʹ- AACCGATTCCGCACAGTCTT-3ʹ.

### Genotyping

Tail DNA was prepared using the following procedure adapted from a previously described method [20]. Mice were genotyped using PCR QuantStudio 3 realtime PCR system (Thermo Fisher Scientific) with melt curve analysis. Primers for *Cre* and *Adam17* are as previously described [21,22].

### Diet models

Starting at 8–10 weeks of age, mice were maintained on a chow diet (Teklad 7019) or started on a HFD (60% kcal%, D12492, Research Diet Inc.). Baseline body weights were recorded, and subsequent body weights determined at indicated weeks of diet feeding. For HFD, 3–4 food pellets were maintained on the cage floor to encourage sustained weight gain. Body weights were tracked for indicated durations of diet feeding.

### Whole-body metabolic phenotyping

The PhenoMaster System (TSE Systems) allows long-term measurement of multiple parameters within the mouse home cage. Home cage measurements included indirect calorimetry (pull mode), body weight, food and water intake and activity (X, Y, Z: IR beam-break). Total energy expenditure (EE), resting energy expenditure (REE), and metabolic substrate utilization (respiratory exchange ratio, RER = volume CO_2_ produced/volume O_2_ consumed) were calculated from the indirect calorimetry data (oxygen consumption and carbon dioxide production). The system consisted of 12 separate home cages which housed an individual mouse for testing. The home cages were contained in a climate-controlled chamber which allowed manipulation of temperature (4–35°C), humidity, and the light/dark cycle. For indirect calorimetry measurement, the PhenoMaster was run in continuous mode (1 box for each sensor) with a reference sample (volume matched tube with room air) interval of 26 min and baselining acquisition of 2 min. This interval ensured appropriate baselining of individual cage gas sensors with a loss of 2 min of gas measurement for every 24 min of recording time. All other sensors (feed, drink, body weight, activity) were baselined prior to each measurement run. Gas sensor calibration was performed before each measurement session and consisted of a two-point calibration for the oxygen sensor (20%vol and 20.9%vol) and three-point calibration for the carbon dioxide sensor (0%vol, 0.05%vol and 0.95%vol). Binary file write intervals were set to 5 s, flow rate (pull-mode) was set to 0.35 L/min with analog-to-digital smoothing of 10 s, and reference measurement instead of the animal box every 12 cycles. Calculations for VO_2_ and VCO_2_ were set to use the constants 3.941 and 1.106, respectively, giving the following form of the Weir equation, EE = 0.06*(3.941*VO_2_ + 1.106*VCO_2_), where EE is in kilocalories per hour, and VO_2_ and VCO_2_ are in millilitres per minute. Activity measurement scan rate was set to 100 Hz with a recording interval of 1 min and refractory period of 0.8 s. For food and water sensors, ADC smoothing was set to 5 s and a binary data write interval of 10 s. For body weight measurement, ADC smoothing was set to 15 s and a threshold of 15 g for sample measurement. The climate control chamber was set to 50% relative humidity, 23°C for temperature (unless otherwise noted), and a light intensity of 70%. TSE PhenoMaster V6.7.0 was used for data acquisition and data export.

Indirect calorimetry values were exported in a form which was not corrected for body mass. These values correspond to VO_2_ [3], VCO_2_ [3], and EE, denoted as H [3] in the PhenoMaster program. Additional measurements exported included XT+YT, XA, YA; RER, Drink, Feed, and Weight. All values were selected in the ‘View’ menu, the ‘Export table’ setting was set to ‘Format 1’ and data exported via the ‘Export’ menu and navigation to the Export > Table and setting ‘Save as type’ to ‘.CSV’. After data export, data was uploaded to the web-based indirect calorimetry analysis tool, CalR, located at https://CalRapp.org/. CalR imports raw data files, generates plots, and determines the most appropriate statistical tests for interpretation. Analysis using the generalized linear model (which includes ANOVA and ANCOVA) allows for flexibility in interpreting diverse experimental designs, including those of obesity and thermogenesis. All mice were acclimated to single housing for 5 days as well as 2 days in the metabolic phenotyping cages prior to measurements being taken.

### IPGTT

Glucose tolerance testing was performed on indicated mouse strains as described in text and figure legends. Mice were fasted overnight for approximately 16 h by transferring mice to clean cages with no food but continued access to drinking water. The following day, mice were weighed and approximately 3 mm section of tail removed with sterile surgical scissors immediately prior to glucose tolerance testing. The first drop of tail blood was discarded and a second drop of tail blood was expressed and placed on an AimStrip® Plus Blood Glucose Test Strip (Germaine Laboratories, Inc.) in combination with an AimStrip® Plus Blood Glucose Metre (20 to 600 mg/dL glucose range, Germaine Laboratories, Inc.). This datapoint was the baseline glucose level (t = 0 min). A 10% w/w solution of D-glucose was made by diluting stock D-glucose (45% w/w D-glucose in H2O, G8769 Sigma-Aldrich) in normal saline (114–055-721, Quality Biological). The 10% glucose solution was warmed for 30 min in a 37°C bead bath prior to syringe loading. Mice were injected intraperitoneally (IP) with the volume of 10% w/w glucose injected (μl) = 10 x body weight (g), which is equivalent to 1 g of glucose/kg of body weight. Tail blood glucose levels were measured at 15, 30, 60 and 120 min (t = 15, t = 30, t = 60 and t = 120) after glucose injection. All measurements were made on unanesthetized mice.

### Body composition analysis

Total body composition in mouse carcases was performed using an EchoMRI™-100 H Body Composition Analyser (Echo Medical Systems, LLC) with access to the equipment kindly provided by Stefan Hargett at the University of Virginia.

### Histology

Tissues were harvested immediately from euthanized mice and placed into 10% buffered formalin. Formalin-fixed tissues were processed by the VCU Massey Cancer Centre Cancer Mouse Model Shared Resource for paraffin block embedding, sectioning, and histology slide preparation. All tissues were processed via a Sakura Tissue Tek automated processor using a standard dehydration, clearing, and paraffin infiltration program, and were sectioned at 5 µm thickness. Slides were further processed and stained with haematoxylin and eosin and digitally scanned using the VectraPolaris 1.0 Automated Quantitative Pathology Imaging System (PerkinElmer, Inc.) at a resolution of 0.25 µm/pixel (40X). Slide images were viewed using PhenoChart software (PerkinElmer, Inc.). An unbiased pathologist qualitatively examined the scanned images for adipocyte size as well as mononuclear infiltration as a measure of inflammation.

### RNA-sequencing

All RNA library preparation and sequencing was performed by BGI Americas using the DNBseq high-throughput sequencing methodology that utilizes combinatorial Probe-Anchor Synthesis (cPAS), linear isothermal rolling circle amplification (RCA) and DNA Nanoball (DNB) technology, followed by high-resolution digital imaging [23,24]. In brief, ribosomal RNA was depleted, mRNA was captured using oligo(dT) capture magnetic beads, and the purified mRNA was fragmented and converted to double-stranded cDNA which was end repaired, 3ʹ adenylated and sequencing adaptors ligated to the 3ʹ adenylated fragments. Adaptor containing fragments was PCR amplified, purified, and then converted to single-stranded cDNA circles which were the final format of the sequencing library. This library underwent final linear amplification with phi29 polymerase (Rolling Circle Amplification) to generate 300–500 copies of the library which folded into DNBs and these were loaded onto the BGISEQ-500 platform for sequencing using single-end 50 bp reads. De-multiplexed, adaptor trimmed reads passing quality control were obtained from BGI.

All alignment and differential gene expression analysis was performed using the publicly available, virtual bioinformatics analysis environment Galaxy (https://usegalaxy.org/ [25]). Reads were aligned to the mouse mm10 reference genome using the STAR alignment program [26]. Read counts were then calculated using FeatureCounts [27] using Mus musculus.GRCm38.92 for an annotation file. DESeq2 [28] was then used for differential expression analysis with all samples added to their appropriate groups. Principle component analysis (PCA) was conducted using ClustVis [29]. To determine specific transcriptional profiles for the samples, any transcripts with a normalized read count <1 were set to 1. Differentially expressed genes (DEGs) were then filtered with the criteria that there must be a greater than 2-fold change between groups with an adjusted p-value <0.05. Additionally, normalized read counts for either group in the comparison must be >100. Graphs were generated using GraphPad Prism 8.1.

### Flow cytometry

Following euthanasia, pWAT was excised and weighed. Tissue was then minced into <3 mm pieces using a razor blade. Minced tissue was then rested in DMEM with 1% FBS until all tissue was collected and ready for digestion. Tissue was digested in DMEM with 0.5% FBS and 0.5 mg/mL Collagenase A (Roche) for 45 min at 37°C with thorough vortexing every 10 min. Digested tissue was then strained through a 100 um filter and centrifuged at 300 g for 5 min. The adipocyte cake was removed by vacuum and the cell pellet rinsed with FACS buffer (PBS with 5% FBS and 2 mM EDTA). Cells were then stained for live-dead exclusion using Zombie Aqua (BioLegend, 423,102) according to manufacturer’s protocol. Cells were washed with FACS buffer. Fc receptors were blocked with 5 μg 2.4G2 (130) for 10 min at 4°C. Antibodies were added at indicated concentrations (Supplemental Table 1) for 45 min at 4°C. Cells were fixed in Fixation Buffer (BioLegend, 420,801) for 10 min at room temperature. For intracellular staining, following fixation, cells were permeabilized using Intracellular Stain Permeabilization Buffer (BioLegend, 421,002) according to manufacturer’s protocol. Briefly, cells were stained for intracellular markers for 60 min at room temperature and washed two times with intracellular permeability buffer and then fixed using Fixation Buffer for 10 min at room temperature. For cytokine flow cytometry, single cells were stimulated with 25 ng/mL PMA and 1uM ionomycin in the presence of 1uM Brefeldin A and 1uM Monensin for 3 h at 37°C. Following stimulation, cells were stained for surface markers and intracellular markers as described above. Antibodies used in flow cytometry experiments can be found in Supplemental Table 1. Flow cytometry data were collected on an LSR Fortessa X-20 (BD) and analysed in FlowJo version 10 (BD).

### Statistical analysis

Statistical analyses and graphing were conducted using GraphPad Prism 8.1. All data were analysed with a student’s T test (or non-parametric equivalent) when comparing two groups and one-way ANOVA with Tukey’s post-test (or Brown–Forsythe and Welch ANOVA test for non-parametric data) when comparing more than 2 groups. For body composition experiments, t-tests were used to compare lean body mass between genotypes and also fat body mass between genotypes. Experiments shown were repeated at least twice independently. Significant p-values are noted on figures. If p values are not indicated, the data is not statistically significant.
